# High-throughput sgRNA testing reveals rules for Cas9 specificity and DNA repair in tomato cells

**DOI:** 10.3389/fgeed.2023.1196763

**Published:** 2023-06-06

**Authors:** Ellen Slaman, Michiel Lammers, Gerco C. Angenent, Ruud A. de Maagd

**Affiliations:** ^1^ Laboratory of Molecular Biology, Wageningen University & Research, Wageningen, Netherlands; ^2^ Bioscience, Wageningen University & Research, Wageningen, Netherlands

**Keywords:** CRISPR-Cas9, DNA repair, CRISPR specificity, tomato, protoplast

## Abstract

CRISPR/Cas9 technology has the potential to significantly enhance plant breeding. To determine the specificity and the mutagenic spectrum of SpCas9 in tomato, we designed 89 g(uide) RNAs targeting genes of the tomato MYB transcription factor family with varying predicted specificities. Plasmids encoding sgRNAs and Cas9 were introduced into tomato protoplasts, and target sites as well as 224 predicted off-target sites were screened for the occurrence of mutations using amplicon sequencing. Algorithms for the prediction of efficacy of the sgRNAs had little predictive power in this system. The analysis of mutations suggested predictable identity of single base insertions. Off-target mutations were found for 13 out of 89 sgRNAs and only occurred at positions with one or two mismatches (at 14 and 3 sites, respectively). We found that PAM-proximal mismatches do not preclude low frequency off-target mutations. Off-target mutations were not found at all 138 positions that had three or four mismatches. We compared off-target mutation frequencies obtained with plasmid encoding sgRNAs and Cas9 with those induced by ribonucleoprotein (RNP) transfections. The use of RNPs led to a significant decrease in relative off-target frequencies at 6 out of 17, no significant difference at 9, and an increase at 2 sites. Additionally, we show that off-target sequences with insertions or deletions relative to the sgRNA may be mutated, and should be considered during sgRNA design. Altogether, our data help sgRNA design by providing insight into the Cas9-induced double-strand break repair outcomes and the occurrence of off-target mutations.

## 1 Introduction

CRISPR/Cas-mediated genome editing has rapidly and decisively impacted the field of molecular biology. After the discovery of CRISPR systems functioning as an adaptive immune system in bacteria (Makarova et al., 2006; Barrangou et al., 2007; Brouns et al., 2008; Garneau et al., 2010), they have been engineered into efficient genome editing tools of unprecedented simplicity and speed (Jinek et al., 2012; Cong, Ran, et al., 2013), often replacing pre-existing techniques for inducing targeted mutations, such as those using zinc-finger nucleases or transcription activator-like effector nucleases (TALEN).

CRISPR/Cas-mutagenesis has been successfully applied in many plant species (Jaganathan et al., 2018; Chen et al., 2019; Zhu, Li, and Gao, 2020). The technique has revolutionized plant research and has shown great potential for plant breeding (Lemmon et al., 2018; Zsögön et al., 2018; Kwon et al., 2020). In the European Union, CRISPR-edited crops are subject to the same risk assessment as transgenic crops (referred to as Genetically Modified Organisms -GMOs). This practice was partly motivated by the argument that the technology, unlike other mutagenesis techniques, does not have a long history of safe use.

In the context of CRISPR/Cas-mutagenesis, off-target mutations are induced mutations at positions other than the intended target site. These off-target sites have sequence similarity to the target site and may lead to an unintended disruption of gene function. Some still perceive off-target mutations as a hazard, even if the associated risk is low–especially when compared to spontaneously occurring mutations or genomic changes (Ossowski et al., 2010; Shirasawa et al., 2016; Graham et al., 2020; Bessoltane et al., 2022).

Early studies on CRISPR/Cas9 specificity in mammalian cells revealed that sequences with up to 4 mismatches to the target site could readily be cleaved by Cas9 (Fu et al., 2013; Hsu et al., 2013; Pattanayak et al., 2013). Since then, several studies have been performed on CRISPR/Cas9 specificity in plants, for example, in *Arabidopsis*, rice, maize, cotton, tomato and grapevine (Feng et al., 2014; Q. Zhang et al., 2017; Tang et al., 2018; Lee et al., 2018; J. Li et al., 2018; Wang et al., 2021; Nekrasov et al., 2017; Sturme et al., 2022; Young et al., 2019). These studies suggest that the RNA-guided nuclease activity of Cas9 in plants is mostly specific and off-target mutations seldom occur. In tomato, a large-scale study focused on CRISPR/Cas9-induced mutagenesis of immunity genes found no evidence of off-target mutations in 144 analysed plants (N. Zhang et al., 2020). In an additional study, whole genome resequencing of a mildew-resistant genome-edited tomato was performed. Likewise, no evidence of off-target mutations was found (Nekrasov et al., 2017).

Most studies screening for off-target mutations focus on stably transformed plants. The production of such plants often involve laborious tissue culture and regeneration protocols, which limit the number of sgRNAs that can be feasibly transformed and the number of plants that can be analysed. As a result, it does not allow for a thorough analysis of target/sgRNA-mismatch tolerance or a comprehensive analysis of double-strand break (DSB) repair outcome. Knowledge of mismatch tolerance is not only useful to assess the risk of off-target mutations but is also relevant for designing “multiplexing” mutagenesis experiments, in which highly similar alleles or paralogues are targeted simultaneously, and for allele-specific mutagenesis in heterozygous or polyploid crops. A more comprehensive analysis of DSB repair outcomes increases our understanding of repair mechanisms (in plants) and may increase predictability of mutations. Most published studies on CRISPR/Cas-specificity and DSB repair originate from animal systems, where the use of cell cultures circumvented the limitations of studying whole organisms. Similarly, a more comprehensive overview of CRISPR/Cas9 genome editing specificity and outcomes in plants can be obtained from the study of cell cultures or of cell wall-less protoplasts that can be readily transfected with DNA encoding Cas9 and one or more sgRNAs, or with ribonucleoproteins (RNPs) composed of Cas9 and an *in vitro* produced sgRNA. Like mammalian cell lines, protoplasts allow the screening of sgRNAs in a high-throughput fashion and can give detailed information about both the types and the frequencies of induced mutations when coupled with next-generation sequencing techniques. Here we explore tomato protoplasts for this purpose, employing a flexible 96-well format for protoplast transfection and processing.

For this study, we designed 89 sgRNAs of varying predicted specificities, which together target 30 members of the *MYB* transcription factor gene family in tomato and screened for SpCas9-induced mutations at on-target sites and 224 predicted off-target sites (of which 68 in other *MYB* genes) using next-generation amplicon sequencing. Moreover, we looked at the nature of the induced mutations, predictability of sgRNA efficacy, and the nature and predictability of insertions at the target site. The resulting data provide further insight into the range of mutations induced at on-target sites and the occurrence and frequency of mutations at off-target sites.

## 2 Materials and methods

### 2.1 Selection of sgRNAs and off-target sites

Genes encoding MYB transcription factors in tomato were obtained from the Plant Transcription Factor Database Expression (Jin et al., 2017). A total of 29 MYB genes was selected for sgRNA design (Supplementary Dataset S1). In the selected genes, sgRNAs and potential off-target sites with a maximum of 4 mismatches were predicted using CRISPOR (Concordet and Haeussler, 2018). Potential off-target sites with DNA or RNA bulges compared to the target site are not predicted by CRISPOR and were therefore predicted using Cas-OFFinder (Bae, Park, and Kim, 2014).

### 2.2 Transfection vectors and cloning of sgRNAs

The backbone of the vectors for plasmid transfections was constructed using Golden Gate cloning and parts from the MoClo Toolkit, a gift from Sylveste Marillonnet (AddGene kit #1000000044, and the MoClo Plant Parts kit, a gift from Nicola Patron (Addgene kit #1000000047) (Engler, Kandzia, and Marillonnet, 2008; Weber et al., 2011; Engler et al., 2014). A Level 1 vector for easy cloning of sgRNAs behind an *Arabidopsis thaliana* U6-26 promoter was a gift from Marc Youles (The Sainsbury Laboratory). This vector contains an operon expressing an RFP protein that can be replaced by a spacer in a BsmBI-mediated Golden Gate cut/ligate reaction, allowing pink/white screening of colonies that have successfully integrated the sgRNA (“CRISPRpink.”) To allow its use in a Level 2 vector, the BsmBI sites were replaced by BsaI sites using restriction/ligation. Level 1 constructs pICH4773::proNOS-NPTII-tOCS, pICH4772::2xproCaMV35-Cas9-tNOS, pICH47751::2xproCaMV35S-tGFP-tCaMV35S, pICH54044, pICH54055, the adapted pICH47781::pAtU6-26-CRISPRpink sgRNA expression vector and end-linker pICH41822 were then combined into Level 2 vector pICSL4723.

Subsequently, sgRNA transcription units were completed by replacing the CRISPRpink operon with annealed oligos encoding the spacer in a Golden Gate cut/ligate reaction using BsaI. Oligonucleotide sequences used can be found in Supplementary Dataset S2.

### 2.3 DNA preparation

Highly pure DNA for transfection was prepared from 3 mL of overnight *E. coli* culture in LB medium using the PureYield Plasmid MiniPrep System (Promega), with the following adaptations: bacterial pellets were frozen at −20°C before processing to increase DNA yield, the column was washed twice with the endotoxin removal wash to acquire the desired purity, and plasmid DNA was eluted with 30 µL elution buffer preheated at 60°C.

### 2.4 Protoplast isolation

Tomato (*Solanum lycopersicum*) cv. “Moneyberg” plants were grown *in vitro* on hormone-free ½ concentration Murashige and Skoog medium (Duchefa) with 10 g/L sucrose and 8 g/L plant agar (Duchefa), pH 5.8, at 25°C, under a 16 h photoperiod. *In vitro* grown plants were used for protoplast isolation at 10–12 weeks after sowing. Reagents for protoplast isolation and transfection were prepared according to Sheen (Sheen, 2002). Young, fully developed leaves were cut in a feather-like pattern and incubated overnight in the dark with the abaxial side down in 10 mL digestion solution (0.4 M mannitol, 20 mM MES, 20 mM KCl, 10 mM CaCl_2_, 1% cellulase Onozuka R10 (Duchefa) and 0.25% macerozyme R10 (Duchefa); pH 5.7). The next day, the suspension was gently swirled to release protoplasts. The suspension was sieved through a 100 μm cell sieve into a 50 mL tube. The remaining leaf material was washed with 10 mL W5 solution (154 mM NaCl, 125 mM CaCl_2_, 5 mM KCl, 2 mM MES, pH 5.7) to release additional protoplasts and the suspensions were pooled. The pooled suspension was subsequently centrifuged for 3 min at ×100 g and the pellet was resuspended in 10 mL W5. This washing step was repeated twice, and finally pelleted protoplasts were resuspended in 10 mL magnesium-mannitol solution (0.4 M mannitol, 15 mM MgCl_2_, 4 mM MES, pH 5.7) to prepare for transfection. Protoplast density was determined by counting in a haemocytometer and adjusted to 5.0 × 10^5^/mL.

### 2.5 Plasmid transfection

Protoplasts were transfected in 96 well plates, as described by Wehner et al. (2011), with the following modifications. For each replicate, 5 µL of plasmid DNA (2–3 µg per construct) was transferred to a well of a V-bottom microtiter plate and 30 µL protoplast suspension (containing 1.5 × 104 protoplasts) was added. Transfections were started by adding 35 µL polyethylene glycol solution (40% PEG-4000 (Fluka), 0.2 M mannitol, 0.1 M CaCl2) and mixing by pipetting up and down. After a 10 min incubation, 120 µL W5 solution was added and mixed to stop transfection. Protoplasts were pelleted by centrifuging for 3 min at ×200 g. The pellet was resuspended in 100 µL WI solution (0.5 M mannitol, 20 mM KCl, 4 mM MES, pH 5.7). Protoplasts were incubated in the dark for 24 h at 25°C and GFP expression was imaged 24 h after transfection by confocal microscopy (Leica DM5500) to determine transfection efficiency. Protoplasts were then pelleted by centrifuging at ×200 g for 3 min, supernatant was removed, and pellets were kept at −80°C until DNA extraction. Three biological replicates were performed on separate days.

### 2.6 *In vitro* sgRNA transcription and RNP transfection

sgRNAs were transcribed *in vitro* using the EnGen sgRNA Synthesis Kit (NEB), followed by DNAse treatment to remove the template. The sgRNAs were subsequently purified using the RNA Clean and Concentrator kit (Zymo Research). RNP complexes were formed by combining 1.5 µg (0.5 µL) EnGen SpCas9 NLS (NEB) with 1.5 µg sgRNA in Buffer 3.1 (NEB) in a total volume of 5 μL, and incubated for 20 min at room temperature. The mixture containing RNP complexes was used for the transfection of freshly isolated protoplasts as described previously. Protoplasts were incubated in the dark for 24 h at 25°C, pelleted and kept at −80°C until genomic DNA extraction.

### 2.7 Genomic DNA isolation and amplicon sequencing

Protoplast DNA was purified 24 h after transfection in 96-well format using magnetic beads (NucleoMag Plant, Macherey-Nagel), following manufacturer’s instructions. DNA was eluted in 50 μL, of which 6 µL was subsequently used as a template in 25 µL PCR reactions using PHUSION HotStart Flex DNA polymerase (NEB) to amplify genomic DNA fragments containing target or predicted off-target sites. For the PCR, an initial denaturation for 30 s at 98°C was followed by 38 cycles of denaturation for 10 s at 98°C, annealing for 20 s at 58°C, extension for 20 s at 72°C, and a final extension step of 3 min at 72°C. Primer sequences are listed in Supplementary Dataset S3. The resulting PCR products were visualized on a 2% agarose gel. Equal amounts of PCR products were pooled to obtain sequencing libraries. Libraries were subsequently column-purified with the NucleoSpin Gel and PCR Clean-up kit (Macherey-Nagel), following manufacturer’s instructions. Illumina HiSeq sequencing (paired-end, 2x150 bp reads) was performed commercially by Eurofins Genomics Europe Sequencing GmbH, Constance, Germany.

### 2.8 Sequence analysis

Paired sequencing reads were uploaded to the CLC Genomics Workbench v20, trimmed, merged and demultiplexed using standard settings. Mutation frequencies at target and predicted off-target sites were determined using Amplican (Labun et al., 2019). Amplicons amplified from the genomic DNA of mock-transfected protoplasts were used as controls to normalize on- and off-target mutation frequencies. Mutagenic spectra were determined using a custom Python script for filtering the AmpliCan output and is available from the authors upon request. Large insertions were aligned to the tomato genome and plasmid used for transfection using HiSat2 (Kim et al., 2019).

## 3 Results

### 3.1 sgRNA selection

We selected 29 members of the *MYB* (*MYELOBLASTOMA*) transcription factor gene family as targets. For the selected genes, sgRNAs and potential off-target sites with a maximum of 4 mismatches were predicted using CRISPOR (Concordet and Haeussler, 2018). For each gene, two to six perfectly matching sgRNAs were selected, including at least one with off-target sequences having three or more mismatches (“specific”) and one with one or two mismatches (and 0 mismatches in case of an alternative PAM, “non-specific”). A total of 89 sgRNAs as well as 213 of their respective predicted off-target sites (of which 68 in another *MYB* gene) were selected for the screen. Additionally, we considered potential off-target sites forming DNA or RNA bulges when hybridizing with sgRNAs. These sites are not predicted by CRISPOR and were therefore predicted using Cas-OFFinder (Bae, Park, and Kim, 2014). Several predicted off-target sites were selected where a bulge could not be resolved by an annealing alternative that would result in only a mismatch. This added 11 potential off-target sites to our screen, resulting in a total of 224. An overview of all selected off-target sites can be found in Supplementary Dataset S1.

**FIGURE 1:**
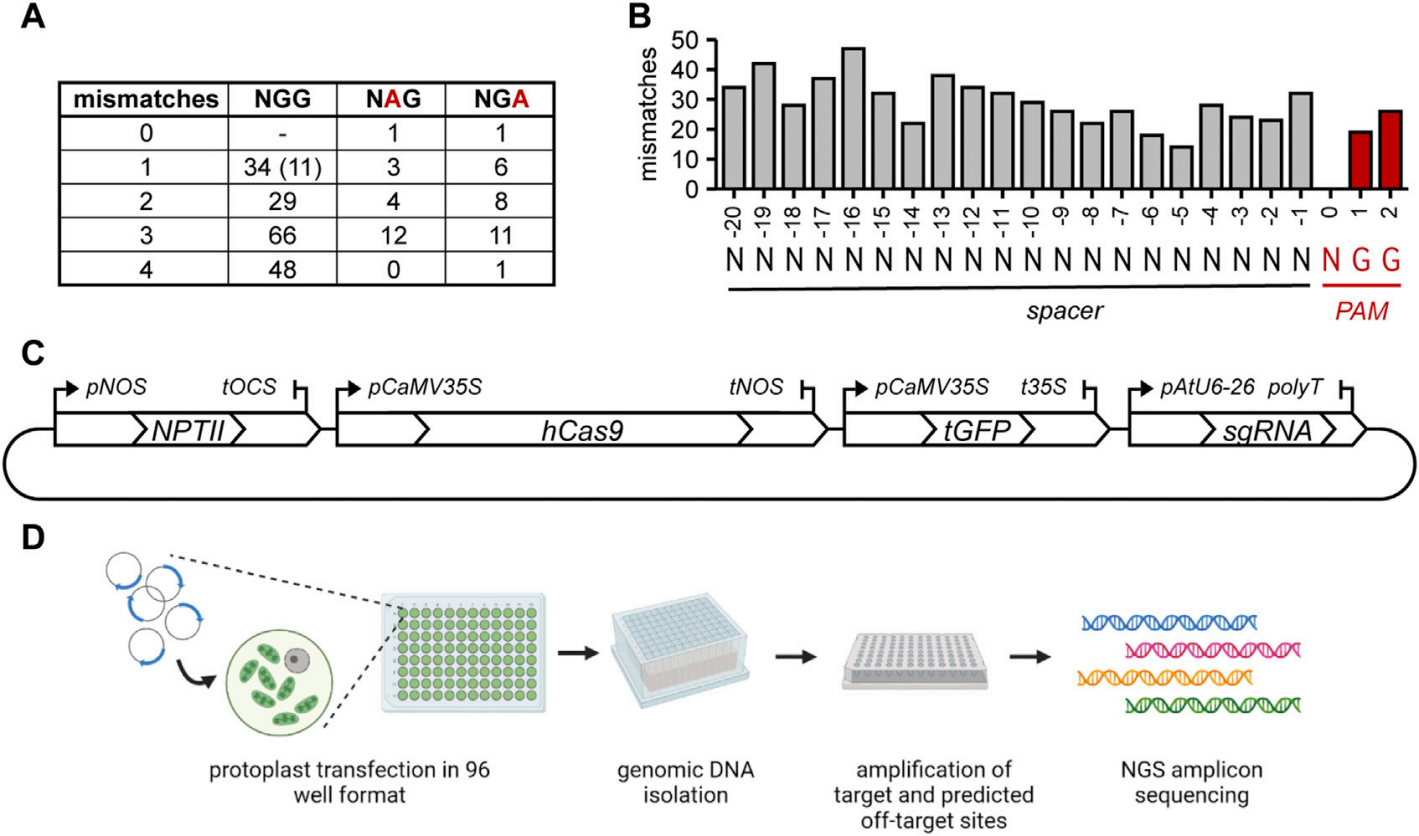
Overview of selected off-target sites and experimental setup. **(A)** number of mismatches and PAM usage in the selected off-target sites. Number between brackets indicates the amount of predicted off-target sites that have an additional insertion or deletion compared to the gRNA in addition to a mismatch. At these sites, sgRNAs can only anneal by formation of an RNA or DNA bulge. **(B)** Distribution of all mismatches in selected predicted off-target sites over the spacer and PAM. **(C)** Graphical overview of the plasmid used for protoplast transfection. **(D)** Overview of the experimental steps. Created with BioRender.com.

Potential off-target sites with 0 (with an alternative PAM) to 4 mismatches to the sgRNA were selected. The alternative NGA or NAG Cas9 PAMs are functional at a low frequency in mammalian cells (Hsu et al., 2013; Cho et al., 2014; Tsai et al., 2015) and in rice (Meng et al., 2017). To further assess to which extent these non-canonical PAMs allow DNA cleavage in plants, we selected several sites that had either an NGA or NAG PAM (Figure 1A). Additionally, the position of the mismatch within the spacer might influence the likelihood of cleavage, resulting in an off-target mutation. Therefore, off-target site sequences with mismatches distributed over the length of the spacer were selected (Figure 1B). Notably, 20 sites were selected that had only a single mismatch in the 12 bp most proximal to the PAM (the so-called “seed sequence”).

### 3.2 CRISPR/Cas9-mediated mutagenesis of tomato protoplasts

Annealed oligonucleotides representing the spacers of the selected sgRNAs were directly ligated in a Golden Gate Level 2 vector containing *SpCas9* and *turboGFP* expression cassettes. This arrangement ensured simultaneous delivery of all components in equimolar amounts (Figure 1C). Thus, the percentages of protoplasts to which *Cas9* was delivered could be determined by fluorescence microscopy and these were found to be similar for the three replicates (41% ± 16%, 41% ± 19% and 42% ± 10%). A graphical overview of the subsequent experimental steps is shown in Figure 1D. Genomic DNA was isolated after 24 h, and fragments containing the target sites and predicted off-target sites were amplified with barcoded primers and sequenced after pooling. Per biological replicate, approximately 7 million pooled paired-end reads were obtained, with a median of approximately 18,200 per sample (average approximately 21,500). The reads were trimmed, merged and demultiplexed and the frequency and type of CRISPR-induced mutations at target and predicted off-target sites were determined.

### 3.3 Correlation between predicted and actual editing efficiencies

**FIGURE 2:**
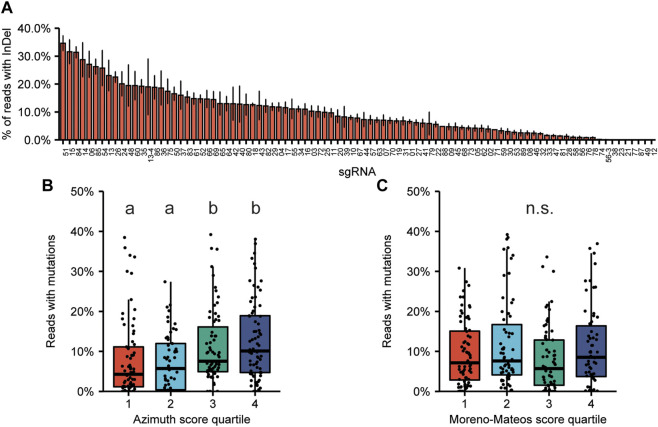
On-target mutagenesis efficiency and correlation with predicted editing efficiency. **(A)** On-target mutation frequencies obtained for the 89 sgRNAs. Sequences of sgRNAs can be found in Supplementary Dataset S1. Error bars indicate SE (*n* = 3). **(B,C)** Distribution of editing efficiencies. The sgRNAs were divided in quartiles based on their Azimuth score **(B)** or Moreno-Mateos score **(C)**. The horizontal line in the box plots indicate the median, boxes represent the second and third quartile, and bottom and top whiskers indicate the first and fourth quartile, respectively. Significant differences between mutation frequencies of sgRNAs in each quartile were determined by Kruskal-Willis test, followed by Wilcoxon’s Rank Sum test for pairwise comparisons.

We did not *a priori* select sgRNAs for high predicted efficiency, and a wide range of mutation frequencies was obtained (Figure 2A). For only 8 targets no mutations could be detected at frequencies at least 0.1% above their wildtype control. The experimentally obtained mutation frequencies were subsequently compared to the predictions as expressed by the Doench score, currently also known as the Azimuth score (Fusi et al., 2015; Doench et al., 2016) and by the Moreno-Mateos score, also known as CrisprScan (Moreno-Mateos et al., 2015). We divided the sgRNAs in quartiles based on their prediction score. For the Azimuth score, experimentally obtained mutation frequencies of sgRNAs in the third and fourth quartile (high score) were significantly increased compared to those in the first and second quartile (Figure 2B). This indicates that the Azimuth score has some, albeit weak, predictive power. No correlation was found between the Moreno-Mateos score and experimentally determined mutation frequencies (Figure 2C).

### 3.4 Mutation types and the predictability of 1 bp insertions following DSB repair

**FIGURE 3:**
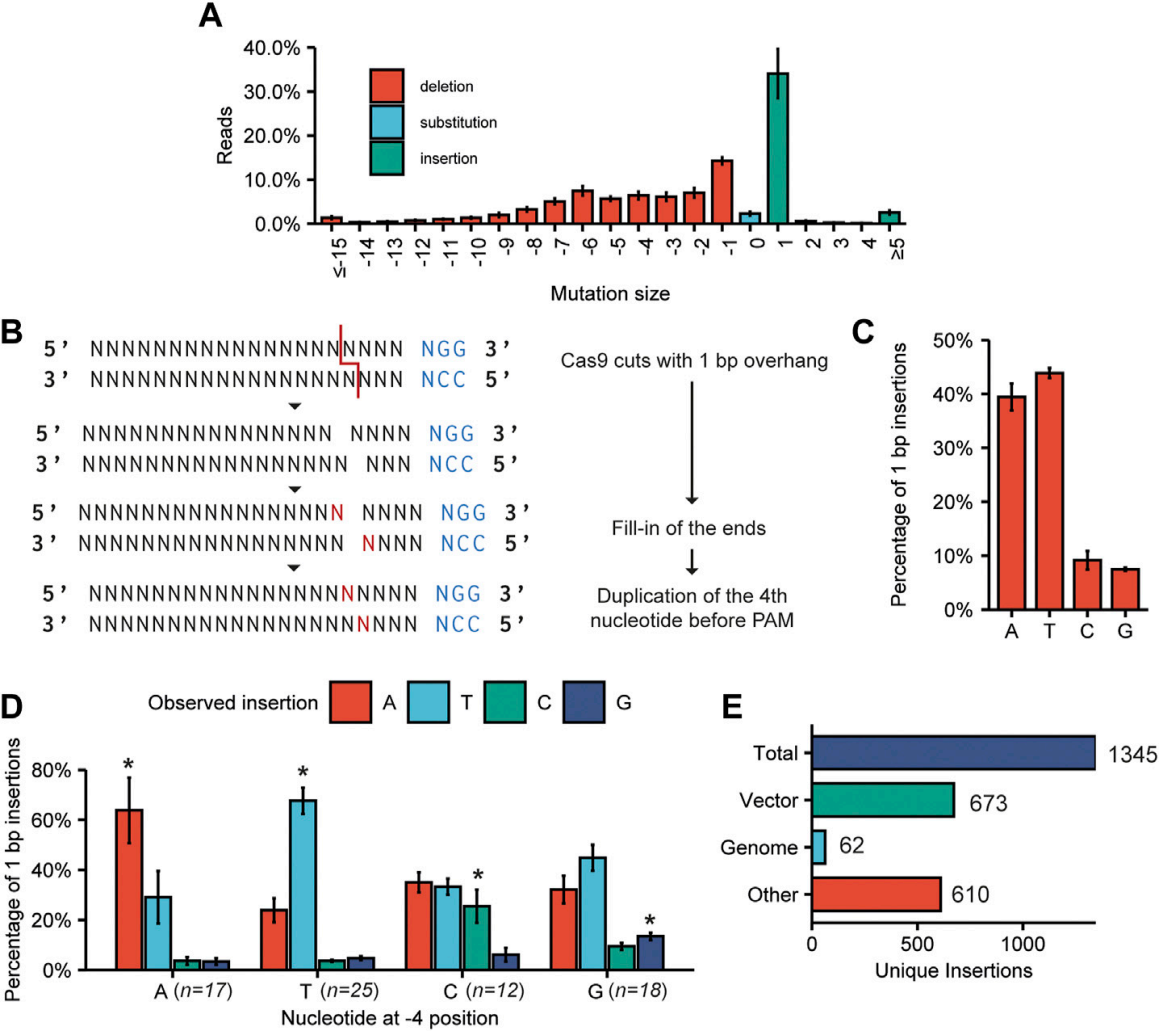
Characterization of mutations found at target sites. **(A)** The compiled spectrum of mutations obtained for all on-target mutations induced by the 89 sgRNAs. The frequency of a mutation with the indicated size was calculated as the percentage of the total number of mutated reads. Error bars indicate SD (*n* = 3). **(B)** Cas9-induced staggered cuts with a 1 bp 5′ overhang can be repaired by templated synthesis and subsequent ligation of the blunt DNA ends. This results in a duplication of the fourth nucleotide before the PAM. **(C)** Frequency of A, T, C, and G insertions at target sites for all sgRNAs. Percentages are calculated by dividing the amount of reads with a certain 1 bp insertion by the total number of reads with a 1 bp insertion. Error bars indicate SD (*n* = 3). **(D)** Percentage of 1 bp insertions in which an A, T, C or G is inserted, observed for targets with an A, T, C or G at the −4 position. Error bars indicate SD (*n* = 3). For statistical analysis, the frequencies of A, T, C, and G insertions were compared between the groups of sgRNAs with either an A, T, C or G at the −4 position. Statistical differences in the 4 groups were determined using one-way ANOVA. Pairwise comparisons were subsequently performed using Tukey’s Honestly Significant Difference *post hoc* test. Significant differences (*p* < 0.05) are indicated by asterisks (*), indicating that a specific insertion occurs more frequently with the particular −4 nucleotide than with any of the other three nucleotides at position −4. **(E)** Origin of large (≥11 bp) insertions. In total, 1,345 unique insertion events (size ≥11 bp) were identified. “Vector” indicates the plasmid used for transfection.

We quantified the types of mutations found at the (on-) target sites (Figure 3A). As seen previously, deletions occurred at a slightly higher frequency than insertions (62% ± 5% and 38% ± 5%, respectively). Most insertions were only 1 bp in size, making that the most common single event, although larger insertions did occur at much lower frequencies. Most deletions were small-sized, and frequencies declined with increasing deletion size. However, larger deletions up to 145 bp were found.

As the 1 bp insertion was the single most favoured mutation induced by Cas9, we investigated this mutation type further. Although it is still often believed that Cas9 introduces breaks with blunt ends, multiple reports state that staggered cuts with 1–3 bp 5’ overhangs frequently occur (Zuo and Liu, 2016; Shou et al., 2018). These overhangs can be filled in by templated DNA synthesis, leading to two blunt ends that can be ligated, resulting in a 1 bp insertion, or more if the overhang is larger (Figure 3B). Thus, in the case of templated insertion, the inserted nucleotide can be predicted from the identity of the −4 position relative to the PAM. This has been shown for yeast (Lemos et al., 2018) and mammalian cells (Shen et al., 2018; Allen et al., 2019; Shi et al., 2019), but not yet for plants.

We investigated all on-target sequencing reads containing a 1 bp insertion. Strikingly, we found a bias for A and T insertions, as opposed to C and G insertions (Figure 3C). Additionally, for every sgRNA, we determined whether the most frequently found insertion at the target site corresponded to the predicted insertion based on the identity of the base at the −4 position. For this analysis, we disregarded sgRNAs that had no or very few (<100) sequencing reads with an identified 1 bp insertion. For only 38 out of the analysed 72 sgRNAs, the most frequently found insertion was equal to the predicted insertion, i.e., a duplication of the −4 base (Supplementary Dataset S4).

Next, all sequencing reads belonging to sgRNAs with the same nucleotide at position −4 were grouped. We then compared the frequency of A, T, C, and G insertions within these groups to the frequencies of the same insertion in the other groups (e.g., the frequency of an A insertion when A is at the −4 position versus the frequency of an A insertion when T, C or G is at the −4 position) (Figure 3D). This revealed a bias towards duplication of the −4 nucleotide for each of the four nucleotides. Interestingly, only for gRNAs that were predicted to result in an A or T insertion was the most frequently observed insertion equal to the predicted insertion. Although C and G insertions occur more frequently for gRNAs with that base at the −4 position compared to the complete set of targets, the most frequently occurring inserted nucleotide was still A or T. We investigated if the bias could be caused by a higher likelihood of Cas9 inducing staggered cuts if the nucleotide at the −4 position is an A or T. This would lead to a higher apparent percentage of 1 bp insertions out of total mutations for sgRNAs with an A or T at the −4 position. However, upon closer inspection of our data, no such significant difference could be found (Supplementary Figure S1).

### 3.5 Origin of larger insertions at target sites

In our screen of merged paired reads we could also identify larger insertions of up to 133 bp at target sites. For the three biological replicates combined, we considered all identified insertions ≥11 bp and aligned these to both the tomato genome as well as to the sequence of the plasmid used for transfection (Figure 3E). In total, we found that 2.07% of the reads (8,761 reads out of 422,323) with a mutation at an on-target site contained an insertion of ≥11 bp. These reads belonged to 1,345 individual distinct events. Of these identifiable insertion events, 673 aligned to parts of the vector used for transfection and another 62 aligned to the tomato genome. For 610 insertion events, the origin of the insertion remains unknown (Figure 3E). An overview of all large insertions can be found in Supplementary Dataset S5.

### 3.6 Experimental identification of off-target sites

To identify off-target mutations, 224 predicted off-target sites were amplified and analyzed for the presence of InDels using AmpliCan (Labun et al., 2019). Amplicons produced from mock-transfected protoplasts were used as controls for normalizing mutation frequencies. To reliably identify true off-target mutations, excluding any sequences that might result from PCR or sequencing errors, we disregarded off-target sites for which no homogeneous control (no Cas9 added) sequences were obtained. Because it might interfere with the identification of genuine mutations, all sites for which the control showed an apparent mutation frequency over 0.1% were disregarded, which was the case for 30 sites. The remaining sites were considered to be a confirmed off-target site if the average mutation frequency was 0.1% or higher and if the mutagenic spectrum showed a pattern expected for CRISPR mutations (e.g., InDels instead of substitutions, as we considered the latter to be more likely due to polymerase or sequencing errors). Mutation patterns at the identified off-target sites can be found in Supplementary Figure S2.

**FIGURE 4:**
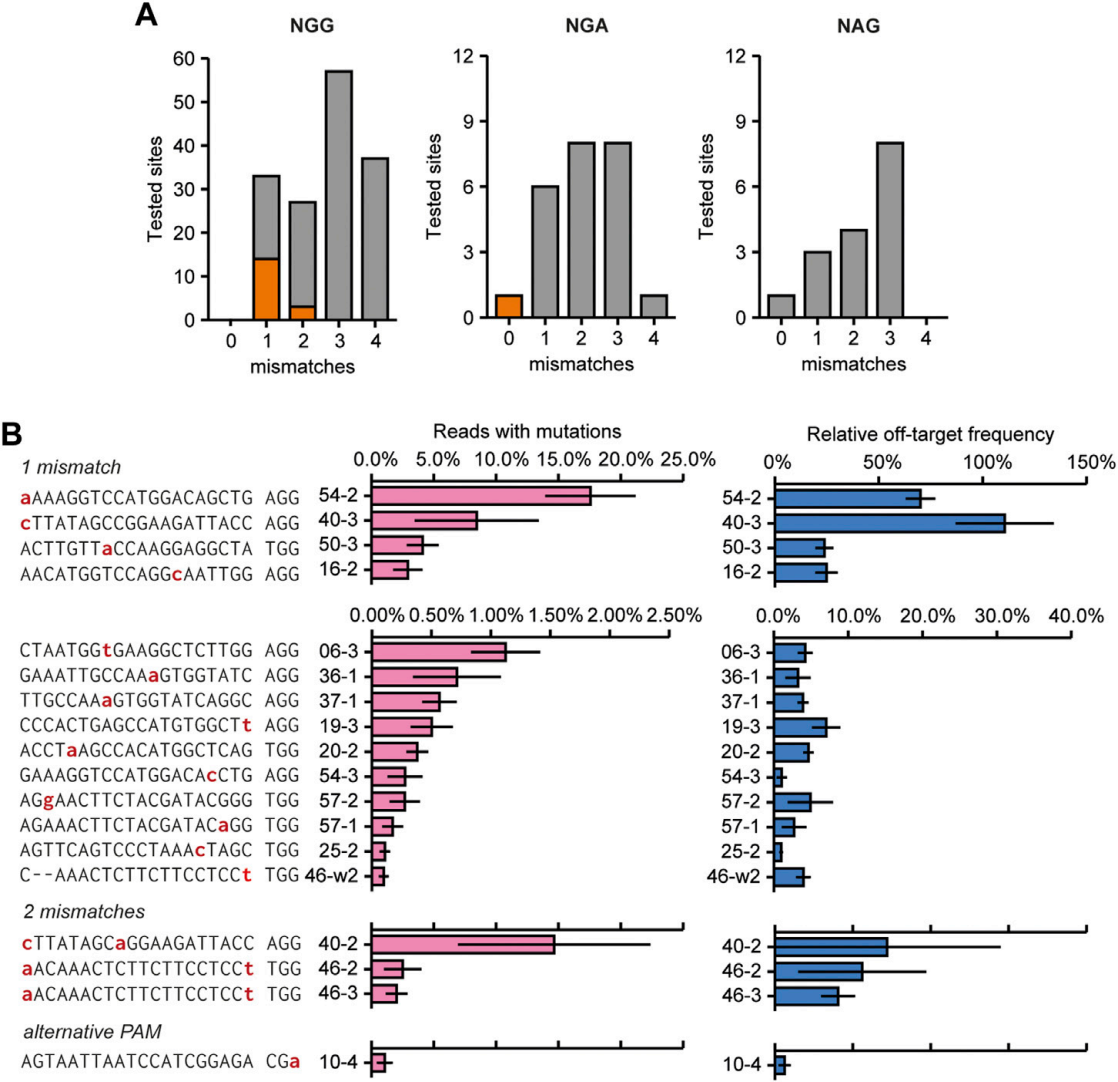
Overview of experimentally confirmed off-target sites identified by screening 226 predicted off-target sites for the presence of mutations. **(A)** Overview showing the number and characteristics of screened predicted off-target sites with 1–4 mismatches compared to the target site sequence (grey bars). Orange indicates the number of experimentally identified off-target sites. **(B)** Mutation frequencies and relative off/on-target frequencies at 18 identified off-target sites that were found to be mutated. Off-target sites are named with sgRNA number (see Figure 2A) with an addition to indicate the different off-target sites for that sgRNA. See also Supplementary Dataset S1. Sequencing data from mock-transfected protoplasts was used to normalize mutation frequencies. Red nucleotides indicate mismatches to the gRNA. Off-target mutations were found at sites that had 1 or 2 mismatches to the target site, or made use of an alternative PAM. Relative off-target frequencies as a measure of how often a genome will contain both a target and an off-target mutation. Percentage are calculated by dividing the off-target mutation frequency by the on-target mutation frequency. Error bars indicate SE (*n* = 3).

At 18 of the remaining 194 analyzed sites, corresponding to 13 sgRNAs, mutations above this threshold were identified (Figure 4A; Supplementary Figure S2 for mutagenic spectrum). Off-target activity mostly occurred at sites that had only one mismatch compared to the target (13 out of 42 tested sites), but was also found for three sites (out of 39) that had a 2 bp mismatch with the guide (Figure 4B). Interestingly, some off-target activity was identified for one site that did not contain any mismatches in the spacer, but had an alternative PAM (NGA instead of NGG, Figures 4A, B). No off-target activity was found at 89 and 49 sites that had 3 or 4 mismatches compared to the target site, respectively.

The two sites with the most frequently occurring off-target mutations (sites 54–2 and 40–3) had a single mismatch which occurred at the nucleotide most distal from the PAM (Figure 4B). Interestingly, off-target effects were only found for sites with two mismatches when one was located at the nucleotide most distal from the PAM (Figure 4B). This indicates that this nucleotide in particular adds very little to specificity and a mismatch here will most likely result in off-target activity. Apart from the two off-target sites mentioned above, off-target mutation frequencies were generally low, never reaching more than 5% of total reads, and in most cases no more than 1% of total reads for the respective sites (Figure 4B).

Rather than the absolute mutation frequencies at off-target sites, the ratio of off-target mutation frequency relative to the on-target mutation frequency is likely to be more relevant for practical situations. Relative off-target frequencies varied widely (Figure 4B, note the different scales). Again, these were highest for the two off-target sites that had a single mismatch most distal from the PAM, even reaching a mutation frequency similar to the on-target site for sgRNA40.

### 3.7 Frequencies of off-target mutations produced by pre-assembled ribonucleoproteins

It is sometimes proposed that the use of ribonucleoproteins (RNPs) might decrease the frequency of off-target mutations. For RNP transfection, purified Cas9 protein preloaded with *in vitro* transcribed or synthetic sgRNAs are introduced in the protoplast.

To compare the (relative) off-target mutation frequencies for the two approaches, we synthesized the 13 sgRNAs for which we found off-target activity in our first screen (Figure 4B) by *in vitro* transcription and loaded them onto purified Cas9 protein. We again transfected protoplasts in a 96 wells format with either plasmid DNA or the corresponding RNPs. Transfection efficiencies for the three biological replicates of plasmid transfections were similar (56% ± 7%, 64% ± 3% and 62% ± 1%). We amplified the on-target sites as well as the previously identified off-target sites and subjected the resulting amplicons to next-generation sequencing. After processing of the reads, the absolute and on-target and absolute and relative off-target mutation frequencies were determined.

**FIGURE 5:**
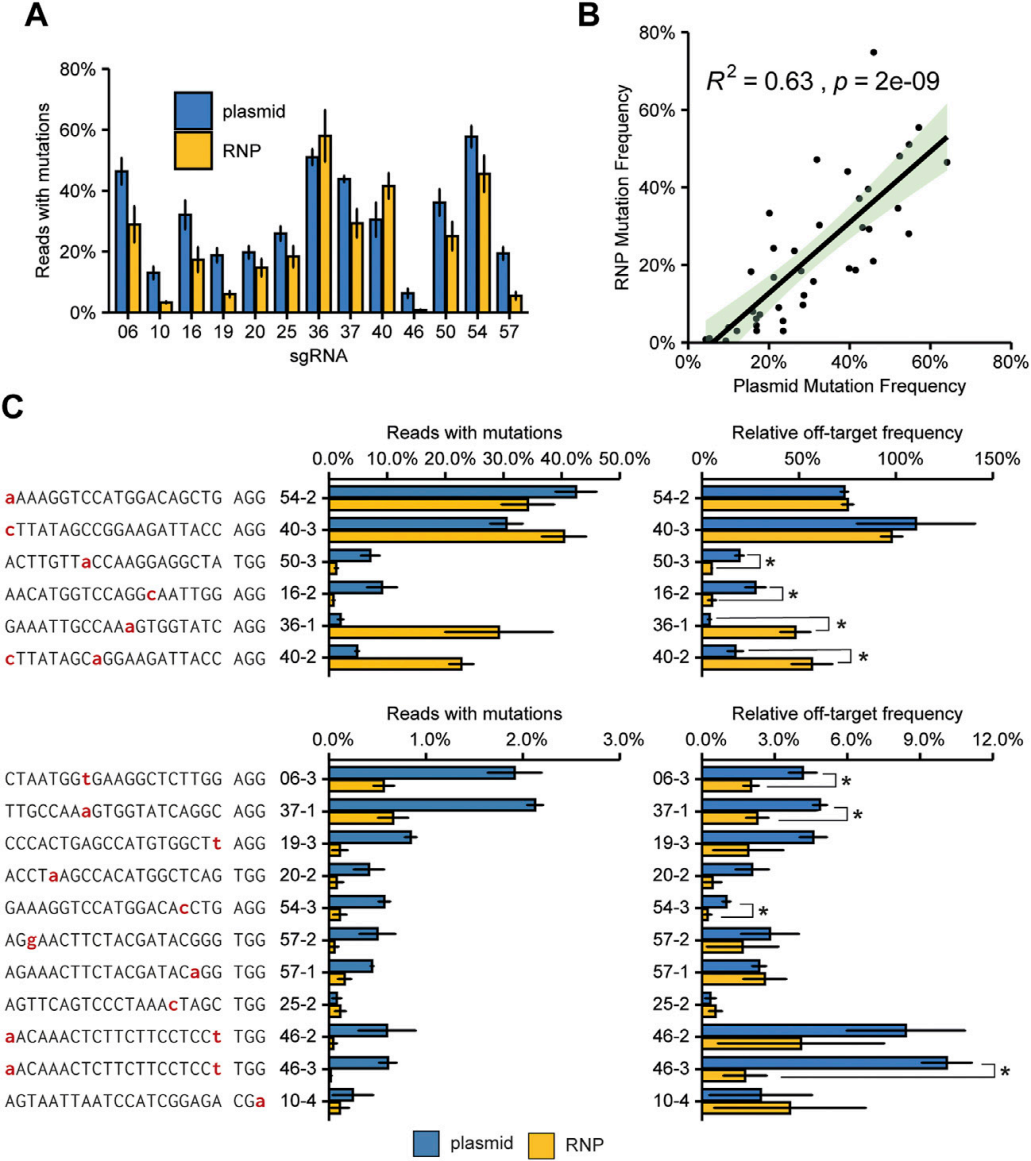
Comparison of plasmid and RNP mediated on-target mutagenesis and off-target mutation frequencies. **(A)** Mutation frequencies at target sites. A total of 15.000 protoplasts was transfected with either 2.5 µg plasmid DNA or 1.5 µg Cas9 enzyme, loaded with 1.5 µg of *in vitro* synthesized sgRNA. Error bars indicate SE (*n* = 3). **(B)** Pearson correlation of mutation frequencies induced by plasmid-encoded Cas9 or RNPs. The green-shaded area indicates the 95% confidence interval. **(C)** Mutation frequencies and relative mutation frequencies at off-target sites. Nucleotides indicated in red indicate a mismatch to the gRNA. Off-target sites are named with sgRNA number (see Figure 2A) with an addition to indicate the different off-target sites for that sgRNA. See also Supplementary Dataset S1. Relative off-target frequencies were determined by dividing the mutation frequency at the off-target site by the mutation frequency at the on-target site. Error bars indicate SE (*n* = 3). Asterisks (*) indicate significant differences (*p* < 0.05), as determined by two-sided Student’s t-test.

Overall, the on-target mutation frequency of plasmid-transfected protoplast was approximately 50% higher than in the first experiment (Figures 2–4), matching the higher transfection efficiency in the latter experiment. The on-target mutation frequencies for plasmid- and RNP-transfected protoplasts were comparable for each target, with less active guides tending to give higher frequencies with plasmid transfection (Figure 5A). Overall, the on-target mutation frequencies for the two methods correlated well (Figure 5B). For most of the tested off-target sites, the use of RNPs either did not significantly affect the mutation frequency or decreased the mutation frequency (Figure 5C). There were two notable exceptions: off-target sites 36–1 and 40–2. At these sites, the RNP complex induced significantly more InDels than Cas9 and sgRNA expressed by the plasmid.

Differences in on- and off-target mutation frequencies between these two types of transfection might simply be explained by varying final amounts of Cas9 protein and sgRNA present in the cells. Therefore, as discussed above for between-sgRNA comparisons, a fairer comparison between plasmid- and RNP-based transfections is the comparison of the ratio of on/off-target mutation frequencies (Figure 5C). The use of RNPs significantly reduced the relative off-target mutation frequency for 6 out of 17 sites. For the two previously mentioned sites 36–1, and 40–2, however, RNPs led to significantly higher relative frequencies. For these two sites, the off-target mutation frequency induced by RNPs was significantly higher than for plasmids (Figure 5C, left panel). Although the on-target efficiency was also higher for RNPs than for plasmids, this difference was much smaller (Figure 5B). For 9 sites, no significant difference in the ratio of on/off-target mutation frequencies was found.

### 3.8 The use of *in vitro* transcribed sgRNAs in RNPs leads to low-frequency incorporation of residual dsDNA template

**FIGURE 6:**
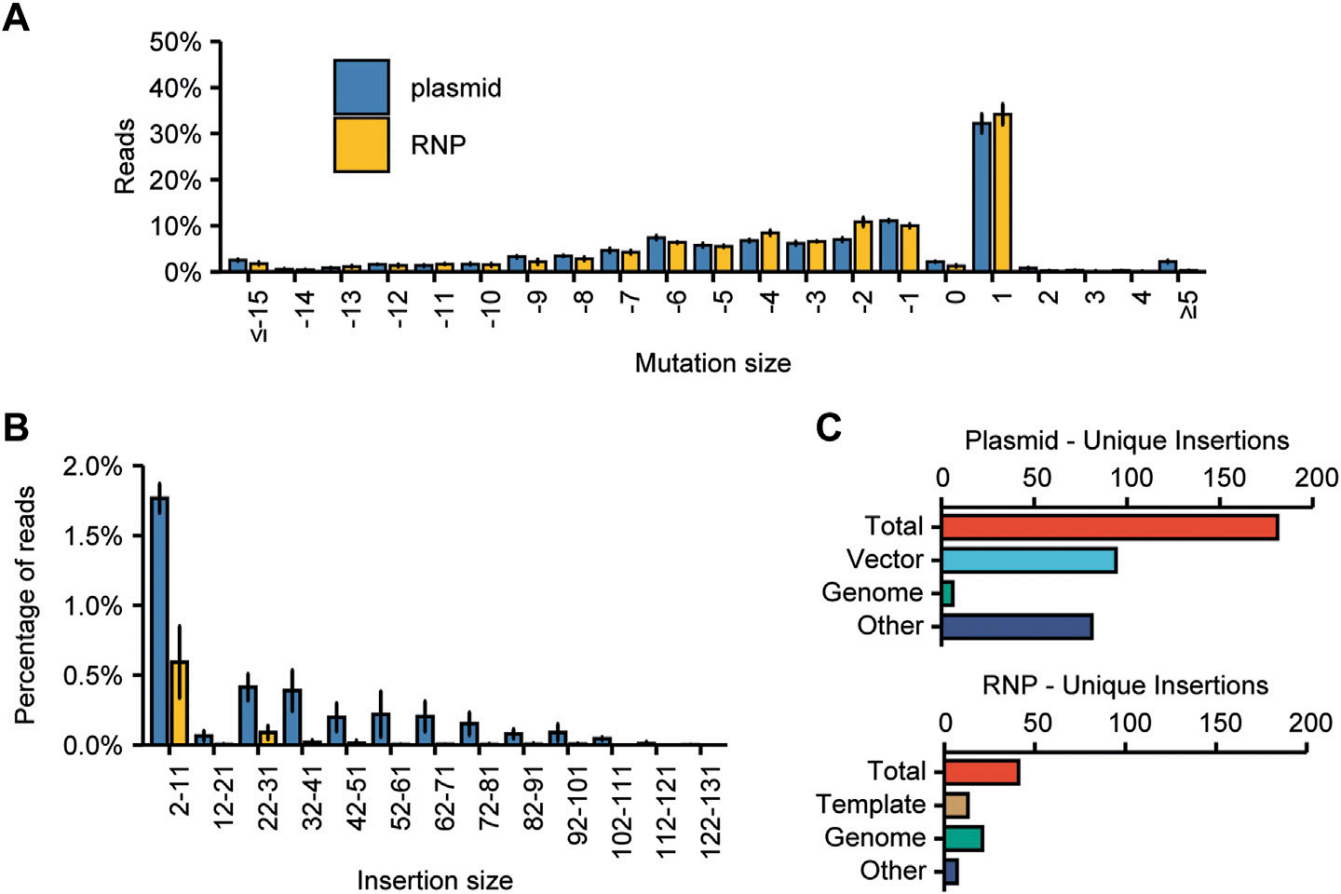
Comparison of the effects of plasmid or RNP-mediated mutagenesis on the frequency and origin of large insertions found at target sites. **(A)** Spectrum of mutations induced by RNPs or plasmid-encoded Cas9. The frequency of an InDel of the indicated size was calculated as the percentage of the total number of mutated reads. Error bars indicate SD (*n* = 3). **(B)** Comparison of the prevalence of insertions of indicated sizes. Percentages are calculated by dividing the number of reads per bin by the total number of mutated reads. Error bars indicate SD (*n* = 3). **(C)** The origin of large (≥11 bp) deletions for plasmid and RNP-transfected protoplasts. “Vector” indicates that the origin of the inserted DNA is the plasmid used for transfection. “Template” indicates that the origin of the inserted DNA is the dsDNA template used for *in vitro* sgRNA synthesis.

It was expected that the use of RNPs instead of plasmid DNA for mutagenesis would reduce the risk of integration of non-host DNA fragments in CRISPR-induced DSBs, as no plasmid DNA is introduced in the cells. To test this, we first determined the spectrum of CRISPR-mutations induced by plasmid-encoded Cas9 and RNPs at on-target sites (Figure 6A). As expected, RNP-induced mutations were very similar to those from plasmid-encoded Cas9, but the percentage of reads that contained insertions larger than 5 bp, although overall low, differed substantially (Figure 6B). A significantly higher percentage of reads from plasmid-transfected protoplasts contained larger insertions, while only very few reads from RNP-transfected protoplasts contained an insertion of 32 bp or larger. In this experiment, we found that for plasmid-transfected protoplasts, 0.86% of on-target reads contained an insertion larger than or equal to 11 bp, pertaining to 181 individual events. For RNP-transfected protoplasts, this number was much lower, at 0.14% and 41 insertion events.

As these large insertion events still occurred, even for RNP-transfected protoplasts albeit a lower frequency, we again attempted to identify their origin. For the plasmid-transfected protoplasts, 94 insertion events out of 181 could be aligned to vector DNA. Another 6 could be aligned to the tomato genome–for the remaining 81 large insertion events, the origin remains unknown (Figure 6C). The relative frequencies for these origins are very similar to those observed in the larger initial experiment (Figure 3E). Interestingly, for the RNP-transfected protoplasts, 13 out of 41 insertion events could be traced back to the dsDNA template that was used to synthesize the sgRNA *in vitro*–even though the resulting sgRNA was treated with DNase. Another 21 events could be aligned to the tomato genome. For 7 events, the origin could not be determined (Figure 6C).

## 4 Discussion

In recent years, the CRISPR/Cas9 system has proven itself to be a valuable tool for genome editing in tomato and other plant species (Zhu, Li, and Gao, 2020; Gao, 2021). However, information about the occurrence of off-target activity in plants, in contrast to animal cells, is limited (Sturme et al., 2022). In this work, we performed a large CRISPR/Cas9 mutagenesis screen with 89 sgRNAs in tomato protoplasts to characterize on-target mutations and quantify off-target mutations. The acquired data give us more insight into the spectrum of mutations formed following Cas9 action and help provide guidelines to prevent off-target action in mutagenesis experiments.

Working with tomato protoplasts allowed us to significantly increase the speed and throughput of our experimental work, in comparison to mutagenesis in regenerating plants. We selected sgRNAs that targeted the coding sequences of 29 genes in the SlMYB transcription factor gene family, in order to simulate a functional screen of MYB functions in tomato. On- and selected off-target mutation frequencies were determined using next-generation sequencing of amplicons. Amplicon sequencing, yielding thousands of sequences per interrogated site, offers improved sensitivity over other methods used to determine mutation frequencies, such as restriction-enzyme polymorphism analysis, T7 endonuclease I assays and Sanger sequencing (Kosicki et al., 2017; Nadakuduti et al., 2019). However, targeted sequencing of preselected amplicons holds some limitations. Off-target mutations at sites that are not predicted by prediction algorithms will not be identified. Moreover, small amplicons of a maximum length of 270 bp were used for sequencing, meaning that large deletions that disrupt primer binding sites could not be detected.

We first investigated mutations induced by Cas9 at target sites. During the selection of the sgRNAs, predicted efficiency was not considered and thus a wide range of mutation efficiencies was acquired (Figure 2A). We attempted to correlate measured efficiency to the efficiency as predicted by the Azimuth score (formerly known as Doench score) and Moreno-Mateos score (Fusi et al., 2015; Moreno-Mateos et al., 2015; Doench et al., 2016). For the Azimuth score, the predicted efficiency had some value (Figure 2B), but no correlation could be found for the Moreno-Mateos score (Figure 2C). Generally, this indicates that a high predicted efficiency score is not a guarantee for a well-performing sgRNA, and *vice versa*. A possible explanation for the different performances of these algorithms might be that the Azimuth model is trained on data acquired from human cells expressing sgRNAs under control of a U6 promoter and by lentiviral transduction, whereas the Moreno-Mateos model is further trained on data acquired from zebrafish embryos. In these zebrafish, mutations were induced using RNPs. Our experimental setup, using plasmid-encoded Cas9 and sgRNAs, more resembles the Azimuth setup. It is also not known to what extent the mutation efficiency is influenced by the genomic context, e.g., the chromatin structure around the target, which could affect the predictive value of the scores.

Further investigation of the types of mutations acquired at target sites revealed that deletions occurred at a slightly higher frequency than insertions. Nevertheless, the single-most frequently occurring mutation was a 1 bp insertion (Figure 3A). As Cas9 frequently induces a DSB with a 1 bp overhang, which may be filled in by DNA polymerases before the loose DNA ends are joined again, these insertions may be predictable (Zuo and Liu, 2016; Shou et al., 2018; Shi et al., 2019). We investigated this for the first time on a large scale in plant cells. Overall, A and T insertions occurred at much higher frequencies than C and G insertions (Figure 3B, Zhang et al., 2020). Although we were able to find a bias in repair for all −4 nucleotides, C or G insertions, when predicted, were still superseded by A or T insertions. In yeast however, more than 75% of insertions were of the predicted base (Lemos et al., 2018), and one study in mammalian cells found that in 149 out of 151 the predicted base was the most frequently occurring insertion (van Overbeek et al., 2016). Our findings indicate that in plants, repair pathways promoting A or T insertions are more frequent than in yeast and mammalian cells. Although the mechanism leading to duplication of the −4 base in mammals and yeast thus appears to be also acting in plant cells, an additional, more dominant mechanism leading to A or T insertion, exists. The existing bias might be utilized to design mutagenesis experiments resulting in specific 1 bp insertions, even if not 100% efficient, for example, for the repair of an interrupted open reading frame.

It is known that DNA fragments may integrate in double stranded DNA breaks, probably through templated synthesis following the annealing of one of the free 3’ DNA ends to other DNA (Köhler et al., 1989; Gorbunova and Levy, 1997; Salomon and Puchta, 1998). We were able to identify mutant alleles, such as large insertions, that occur at low frequencies of around 2% of total mutated reads. Although the integration of such fragments of foreign DNA can be considered undesirable in the creation of mutant plants, these events can easily be identified and avoided by routine screening of the on-target mutation by sequencing. Additionally, these findings add to evidence that NHEJ can be harnessed to specifically insert DNA fragments at target sites, as previously described (Li et al., 2016; Lu et al., 2020; Dong et al., 2020), making this a useful alternative to Homology-Directed Repair (HDR) for insertion of new sequences.

Apart from gaining a better understanding of the mutational spectrum of Cas9, we also aimed to assess the range and frequency of off-target mutations. For this purpose, we selected and investigated 224 predicted off-target sites that had 0 to 4 mismatches to the sgRNA and used either the canonical NGG PAM, or an alternative NAG or NGA PAM. The majority of these sites occurred at positions that had only one mismatch with the sgRNA, indicating that off-target sites that only have 1 mismatch are high-risk. For two of the aforementioned off-target sites, the relative mutation frequency was very high–reaching up to 70% of the on-target mutation frequency for 54–2 and up to 110% for 40–3 (Figure 4B). Interestingly, both these off-target sites had a mismatch at the nucleotide most distal from the PAM. As previously reported, a mismatch at this position leads to a high risk of the off-target being cleaved by Cas9 (Fu et al., 2013; Hsu et al., 2013). Relative mutation frequencies for the other identified off-target sites with 1 mismatch were generally lower, rarely reaching above 10%, indicating that the majority of genomes will only contain mutations at the target site. Additionally, we found off-target activity for three sites that had two mismatches to the target. For all three sites, one mismatch was present at the most distal position, again indicating that this nucleotide adds very little to overall specificity. The last off-target site was identified at a position that had no mismatches in the spacer but made use of an alternative NGA PAM. Although cleavage activity at such sites has already been shown in mammalian cells (Hsu et al., 2013; Cho et al., 2014; Tsai et al., 2015), and for a different non-canonical NAG PAM in rice (Meng et al., 2017), activity at NGA sites had not yet been reported in plants. However, both the off-target mutation frequency and relative off-target mutation frequency at this site were very low.

We have also shown that off-target activity can occur at loci that have an insertion or deletion in comparison to the sgRNA, which can still anneal through the formation of an RNA or DNA bulge. Although our screen was limited in this respect, our results indicate that particularly the formation of an RNA or DNA bulge at the end of the spacer might allow cleavage, even in the presence of another mismatch elsewhere in the spacer (Figure 4B). This is not surprising, as it is known that truncated sgRNAs as short as 17 nt can still result in efficient cleavage of the target site (Fu et al., 2014). Popular sgRNA prediction algorithms such as CRISPR-P 2.0 (Lei et al., 2014; Liu et al., 2017) and CRISPOR (Haeussler et al., 2016) do not take off-target sites with RNA or DNA bulges into account. Therefore, it is advisable to additionally check for this type of off-target sites using one that does, such as Cas-OFFinder (Bae, Park, and Kim, 2014).

We found off-target activity for mismatches distributed over the complete spacer (Figure 4B). This shows that a mismatch in the proposed 7–12 bp “seed” region (Semenova et al., 2011; Jinek et al., 2012; Cong, Rann, et al., 2013) directly adjacent to the PAM does not abolish mutagenic activity completely and that such sites should still be assessed for off-target activity, or avoided. This has previously already been concluded for mammalian cells (Hsu et al., 2013; Pattanayak et al., 2013) and similar results have been published for in planta mutations (Raitskin et al., 2019).

We could not identify any off-target activity at positions that had three or four mismatches compared to the target site. One study in Arabidopsis did show cleavage activity at a site that had three mismatches compared to the spacer (Zhang et al., 2017), as did a study in rice (Tang et al., 2018). One possible explanation for this difference is that in these studies, stable transformation was used, resulting in a prolonged exposure of the genomic DNA to Cas9 cleavage. Thus, chances of off-target activity occurring in stably transformed plants may be further decreased by selecting sgRNAs with only predicted off-target sites with four or more mismatches.

While T-DNA containing Cas9 and sgRNAs may be readily segregated out from self-compatible true-breeding cultivars or parent lines of tomato, T-DNA insertion and subsequent removal by segregation is not an option for plants that require vegetative propagation. For these plants, transient expression or transfecting protoplasts with RNP complexes provide a DNA-free mutagenesis system. Additional advantages of the use of RNPs are that the risk of vector-derived insertions in DSBs is negated, and, as RNP complexes might be degraded faster than plasmid DNA, the time in which the genome is exposed to endonuclease activity is reduced. We have shown that RNPs are a viable alternative to plasmid transfection and can induce high mutation frequencies in tomato protoplasts (Figures 5A, B). We also determined relative off-target frequencies at off-target sites for both methods but conclude that overall, there is no bias for more or less frequent off-target activity with either method.

Summarizing, we can propose several rules to achieve specific genome editing in tomato. To remove the risk of off-target mutations occurring altogether, sgRNAs can be chosen that only have predicted off-target sites with four or more mismatches to the target site using off-target prediction software such as Cas-OFFinder (Bae, Park, and Kim, 2014). However, the selection of such sgRNAs might not be possible for every experiment. Relative off-target mutation frequencies indicate that off-target mutations can generally be avoided in stably transformed plants, even if only a 1 bp mismatch compared to the target is present. Possible exceptions are cases where this mismatch occurs very distal from PAM. If no highly specific sgRNA can be selected, testing off-target sites with up to three mismatches is advisable to mitigate the risk. Unintended on-target events, such as plasmid integration or large deletions, can be avoided by diligently sequencing the target and high-risk off-target sites and ensuring that all alleles have been identified.

In conclusion, we have collected useful data on the specificity and mutational spectrum of 89 sgRNAs for Cas9 in tomato protoplasts. In addition to providing information about the pattern of mutations frequently caused by Cas9-mediated mutagenesis, we have presented evidence for the predictability of 1 bp insertions in planta. We screened predicted endogenous off-target sites and found evidence for off-target activity, of which the majority had only one mismatch compared to the spacer. Off-target activity at sites with three or four mismatches compared to the target site was not found. Thus sgRNAs with only such predicted off-target sites can generally be considered safe. Finally, we have shown that off-target sites with insertions or deletions compared to the target site do pose a risk for off-target mutations, especially if the resulting DNA or RNA bulges are formed at the end of the spacer. This risk may be mitigated by utilizing dedicated sgRNA prediction algorithms that take these types of off-target sites into account. Overall, these results can help sgRNA design and aid in fine-tuning mutagenesis experiments to specific desired outcomes.

## Data Availability

The sequence data presented in the study are deposited in the NCBI repository, BioProject accession number PRJNA975109, https://dataview.ncbi.nlm.nih.gov/object/PRJNA975109?reviewer=1dd2462gj3hv51iet39ngrvv3t.
