# Supplementary material for: High-throughput sgRNA testing reveals rules for Cas9 specificity and DNA repair in tomato cells
Source: Front Genome Ed. 2023 Jun 6;5:1196763. doi: 10.3389/fgeed.2023.1196763 (PMC10279869; doi:10.3389/fgeed.2023.1196763)
Supplement: Supplementary file 4 [file Image1.PDF]

**Supplementary Information belonging to the manuscript entitled "High-throughput sgRNA testing reveals rules for Cas9 specificity and DNA repair in tomato cells"**

**In this file:**

Supplementary Figure 1 – Influence of the nucleotide at the -4 position on the percentage of mutant reads with a 1 bp insertion

Supplementary Figure 2 – Variation plots of identified off-target sites

**Provided separately:**

Supplementary Dataset 1 – Overview Targets, Off-Targets and Genes

Supplementary Dataset 2 – Oligo sequences for sgRNA cloning

Supplementary Dataset 3 – Barcoded primer sequences for target and off-target amplification

Supplementary Dataset 4 – Predicted and actual most frequent 1 bp insertion

Supplementary Dataset 5 – Large insertions identified in screen

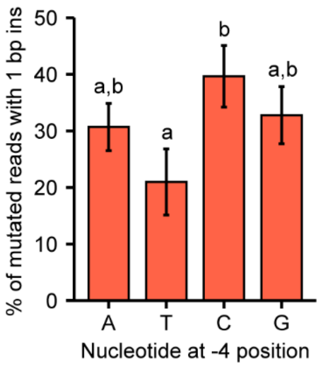


**Supplementary Figure 1. Influence of the nucleotide at the -4 position on the percentage of mutant reads with a 1 bp insertion**. Different letters indicate statistically significant differences between groups, as determined by one-way ANOVA followed by a post-hoc Tukey test. Although A and T insertions occur more frequently overall, the presence of an A or T at the -4 positions does not result in a much higher frequency of 1 bp insertions. This indicates that staggered cuts do not occur more frequently if the -4 base is an A or T.

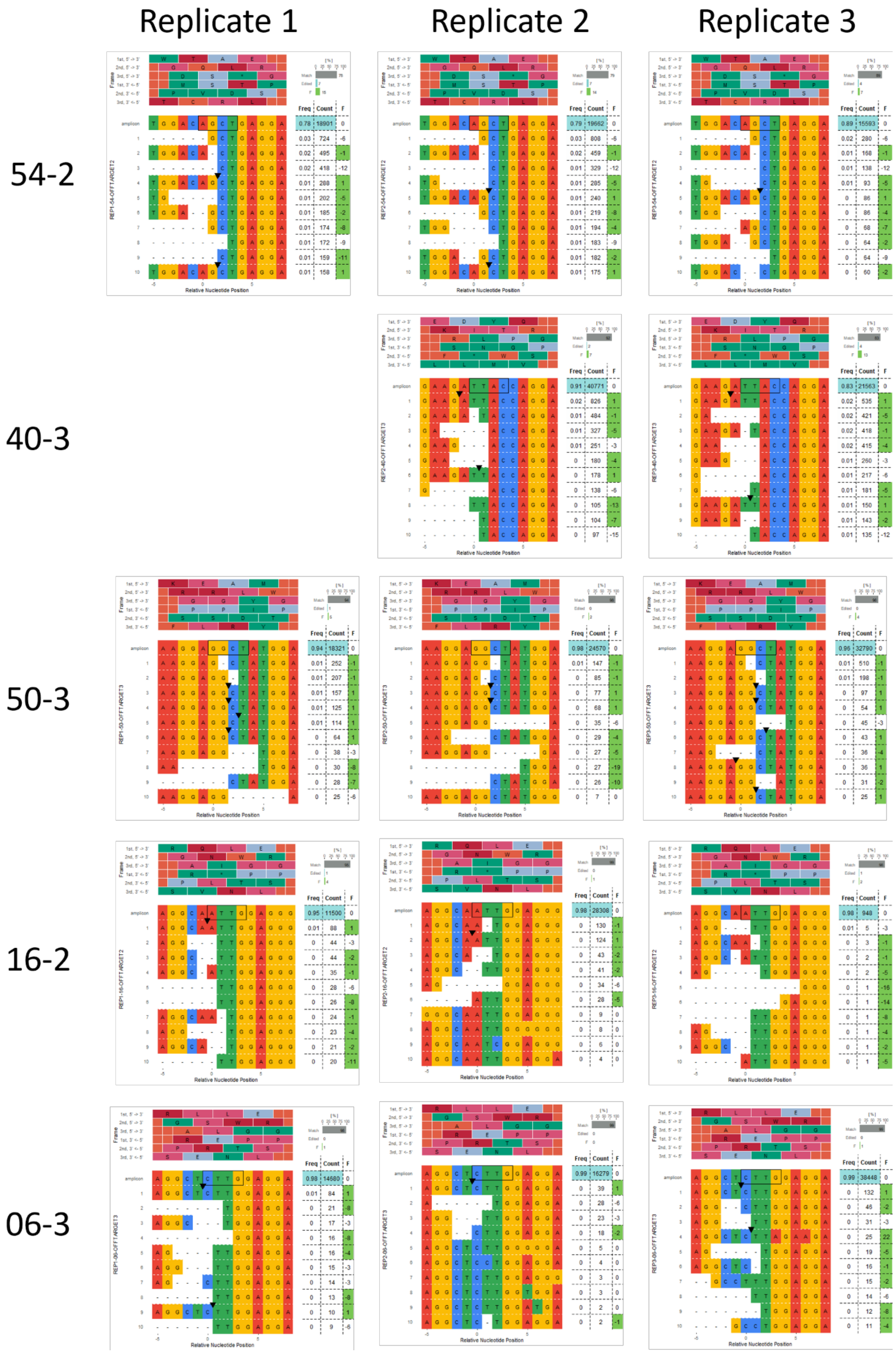
Replicate 1
Replicate 2
Replicate 3
54-2
40-3
50-3
16-2
06-3

| | Replicate 1 | Replicate 2 | Replicate 3 |
|---|---|---|---|
| 36-1 | | | |
| 37-1 | | | |
| 19-3 | | | |
| 20-2 | | | |
| 54-3 | | | |

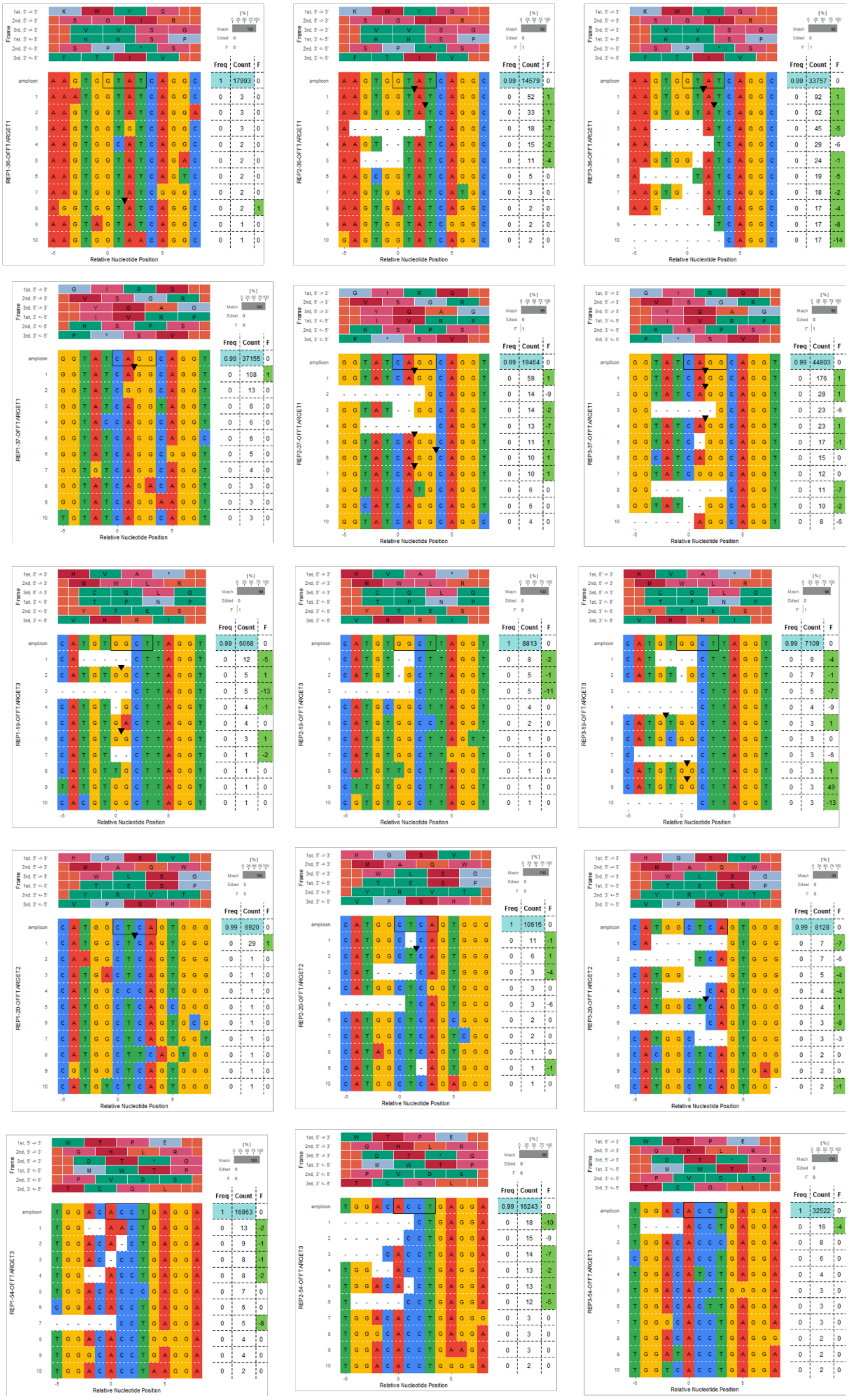

Replicate 1 | Replicate 2 | Replicate 3

57-2

57-1

25-2

46-w2

40-2

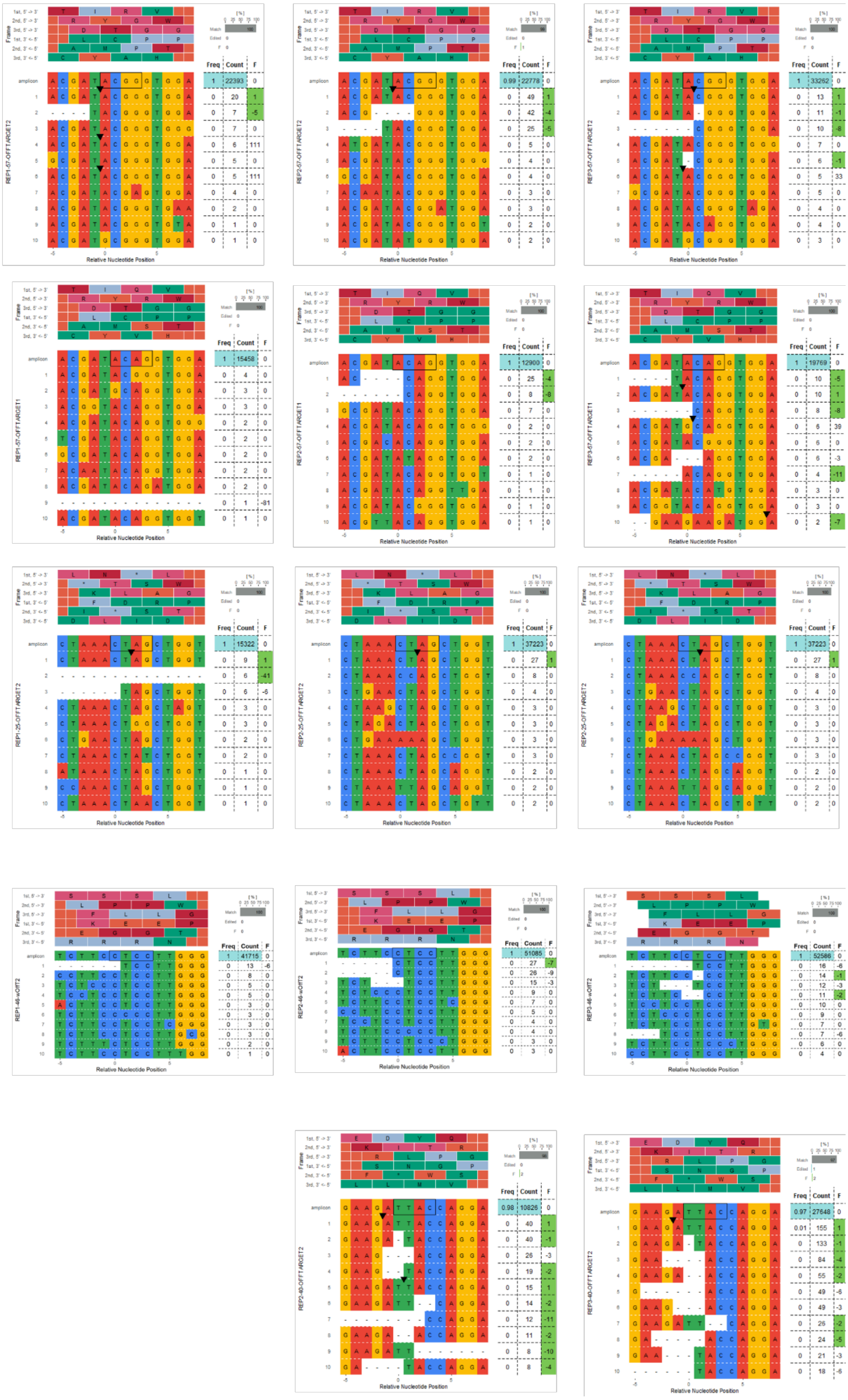

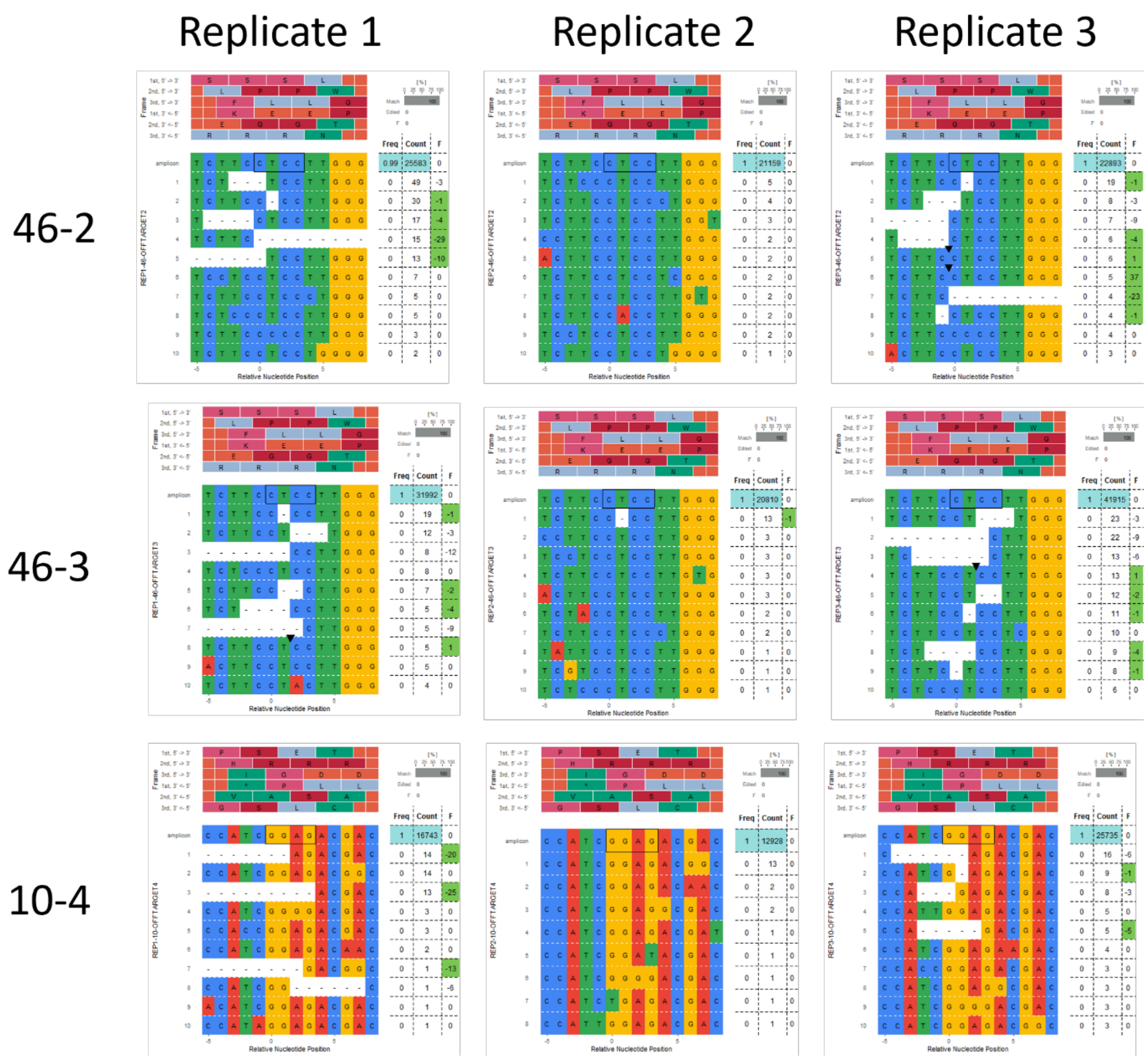


**Supplementary Figure 2. Variation plots of identified off-target sites**. Variation plots for off-target sites were checked to ensure variation was not solely due to sequencing errors, which frequently result in substitutions.
